# Supplementary material for: Oncogenic signaling inhibits c-FLIPL expression and its non-apoptotic function during ECM-detachment
Source: Sci Rep. 2021 Sep 20;11:18606. doi: 10.1038/s41598-021-97715-4 (PMC8452765; doi:10.1038/s41598-021-97715-4)
Supplement: Supplementary file 1 — Supplementary Figures. [file 41598_2021_97715_MOESM1_ESM.pdf]

Supplementary Information

Oncogenic signaling inhibits c-FLIP$_L$ expression and its non-apoptotic function during ECM-detachment

Matyas Abel Tsegaye[1,2], Jianping He[1], Kyle McGeehan[1], Ireland M. Murphy[1], Mati Nemera[1], and Zachary T. Schafer[1,2*]

[1]Department of Biological Sciences, University of Notre Dame, Notre Dame, Indiana 46556, USA

[2]Integrated Biomedical Sciences Program, University of Notre Dame, Notre Dame, Indiana 46556, USA

[*] To whom correspondence should be addressed:

Department of Biological Sciences
222 Galvin Life Science Center
Notre Dame, IN, 46556
Phone: 574-631-0875
Fax: 574-631-7413
Email: zschafe1@nd.edu

A.

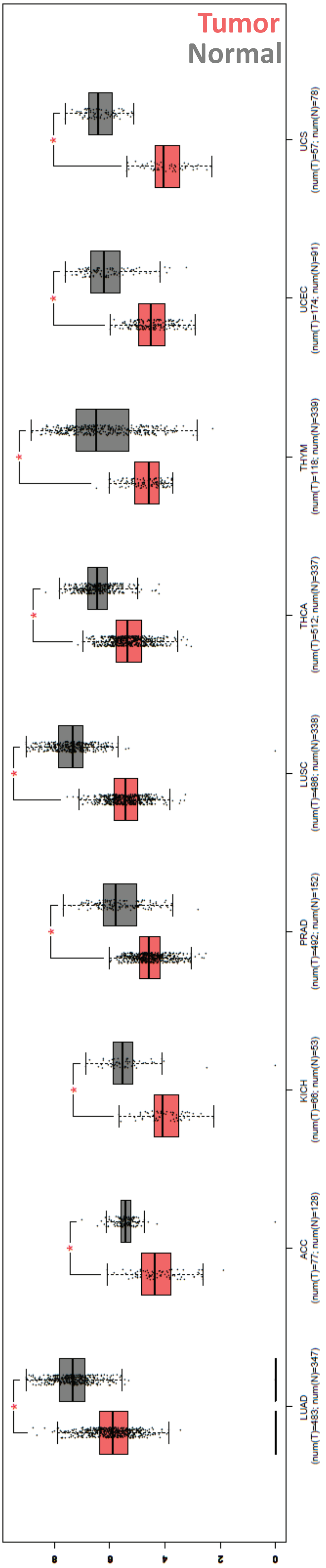

LUAD Lung Adenocarcinoma
ACC – Adenocortical carcinoma
KICH – Kidney Chromophobe
PRAD – Prostate Adenocarcinoma
LUSC – Lung Squamous Cell Carcinoma
THCA – Thyroid carcinoma
THYM – Thymoma
UCEC – Uterine Corpus Endometrial Carcinoma
UCS – Uterine Carcinosarcoma

B.

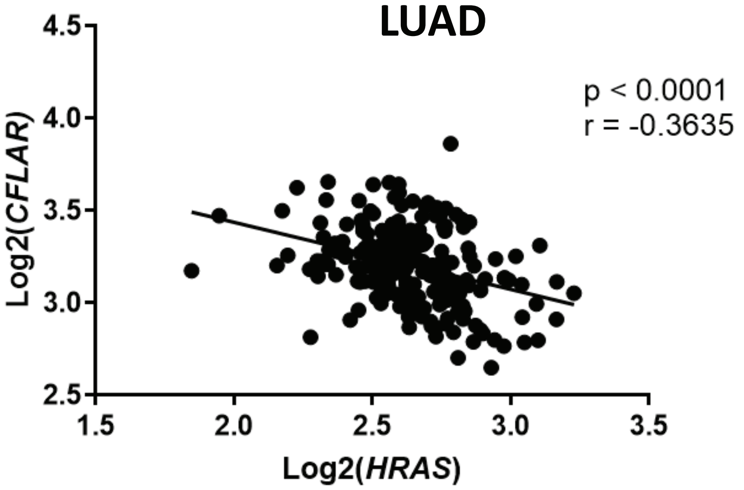

C.

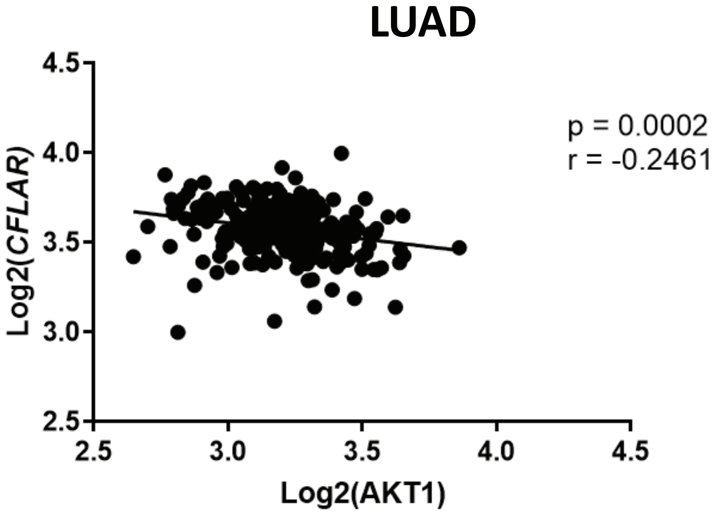

Supplemental Figure 1. Downregulation of CFLAR expression is observed in different cancer types
(A) Comparison of CFLAR expression levels in tumor v sus normal tissue in different cancer types; (B and C) Correlative analysis of the expression levels of CFLAR with (B) HRAS and (C) AKT1 in lung cancer samples.

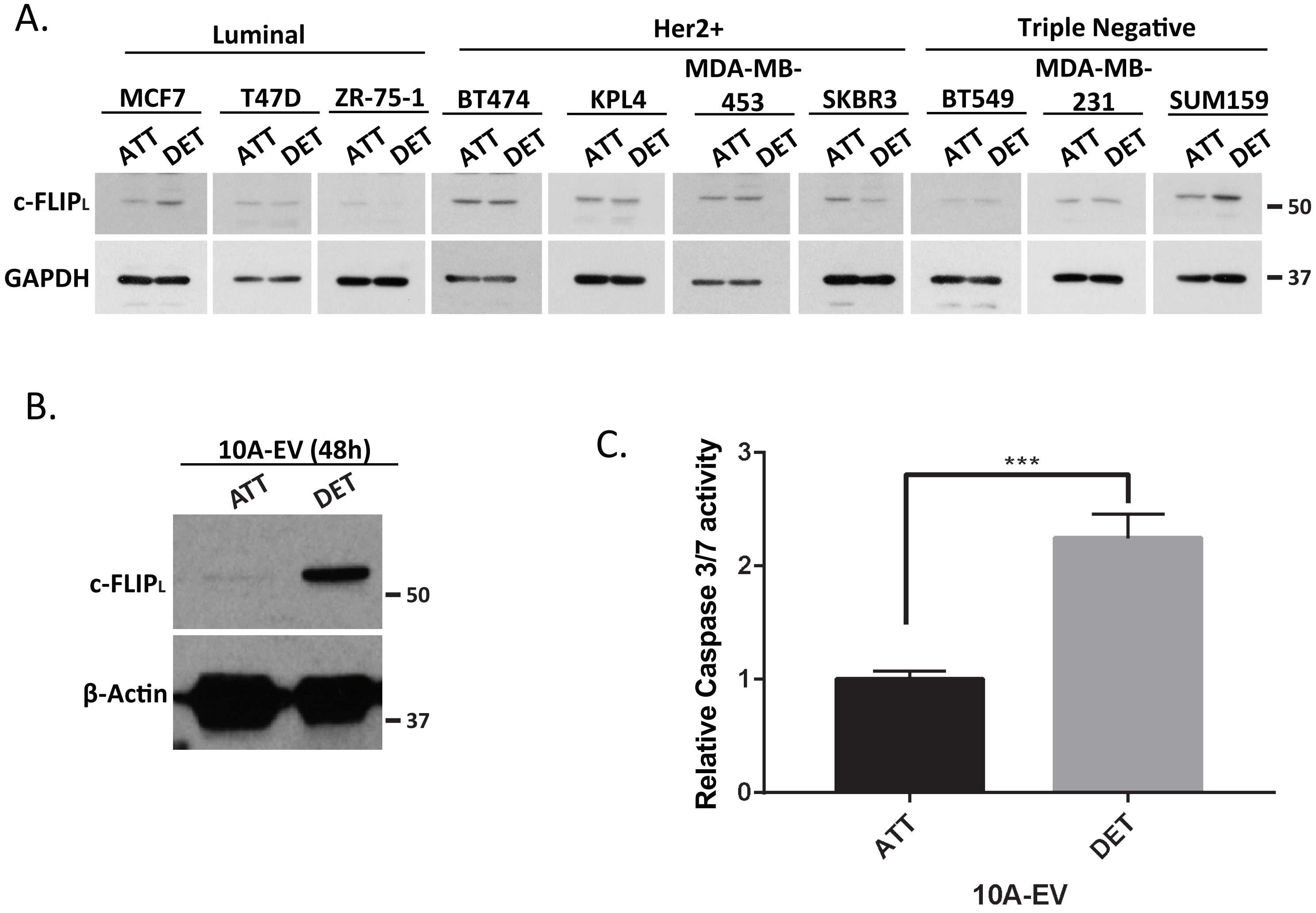

Supplemental Figure 2. Expression of c-FLIP$_L$ during ECM-detachment is not sufficient to block caspase 3/7 activation

(A) Comparison of c-FLIPL protein levels in attachment versus detachment conditions in cell nes representing different subtypes of breast cancer. (B) Measurement of c-FLIPL levels in 10A-EV cells after being grown for 48h in ECM-detached conditions. (C) Measurement of Caspase 3/7 activity in 10A-EV cells after being grown for 48h in ECM-detached conditions. Statistical significance was determined using Student’s two-tailed t-test. Error bars show standard deviation. Western blots and biochemical assays show representative data from three biological replicates. Statistical significance was determined using Student’s two-tailed t-test. Error bars show standard deviation.

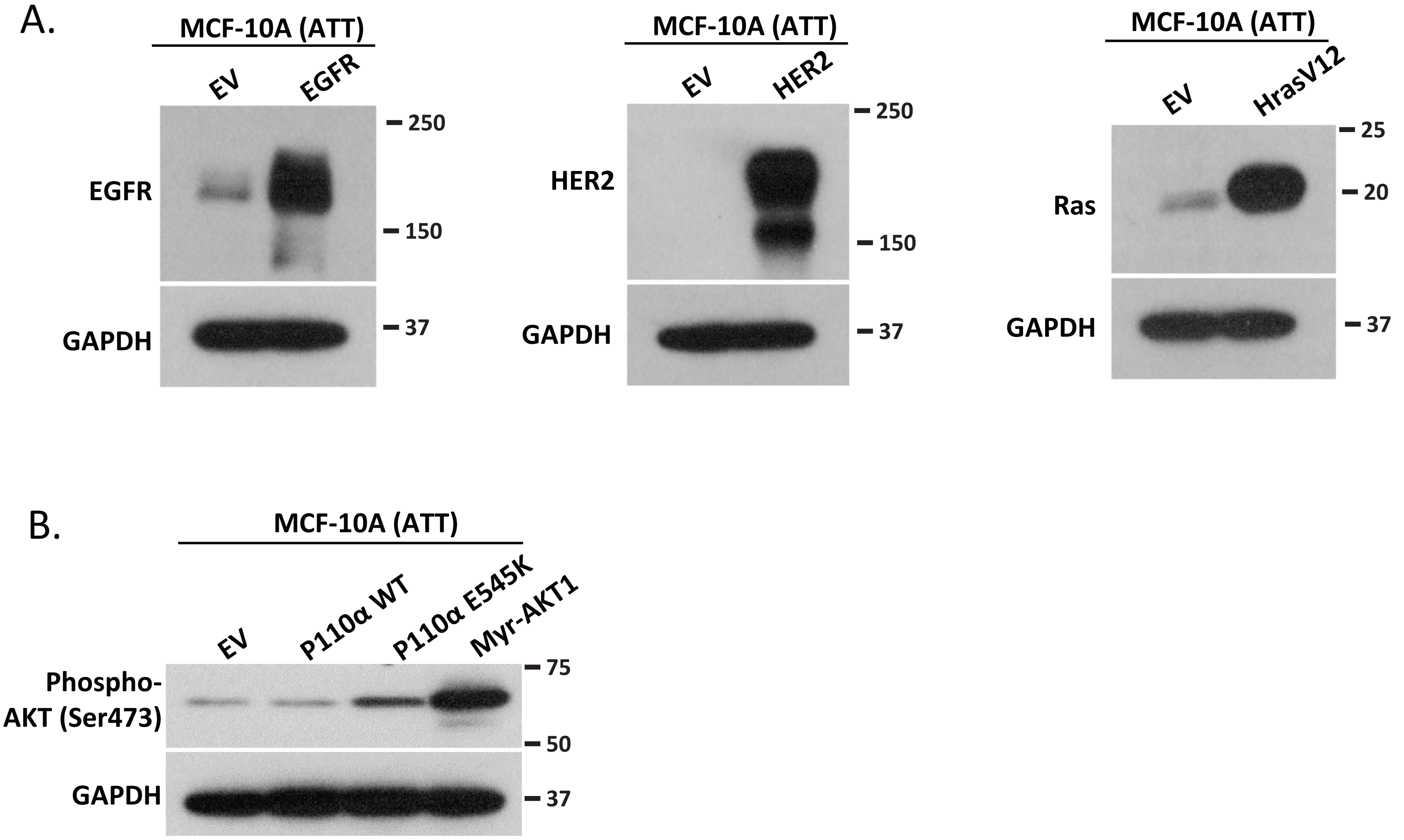

Supplemental Figure 3. Regulation of c-FLIPL is primarily driven by PI(3)K/Akt signaling
(A) Verification of EGFR (left), HER2 (middle), H-Ras G12V (right) overexpression in MCF-10A cells. Confirmation of PI(3)K activation in indicated cell lines via immunoblotting of phopsho-Akt (Ser473). Western blots show representative data from three biological replicates.

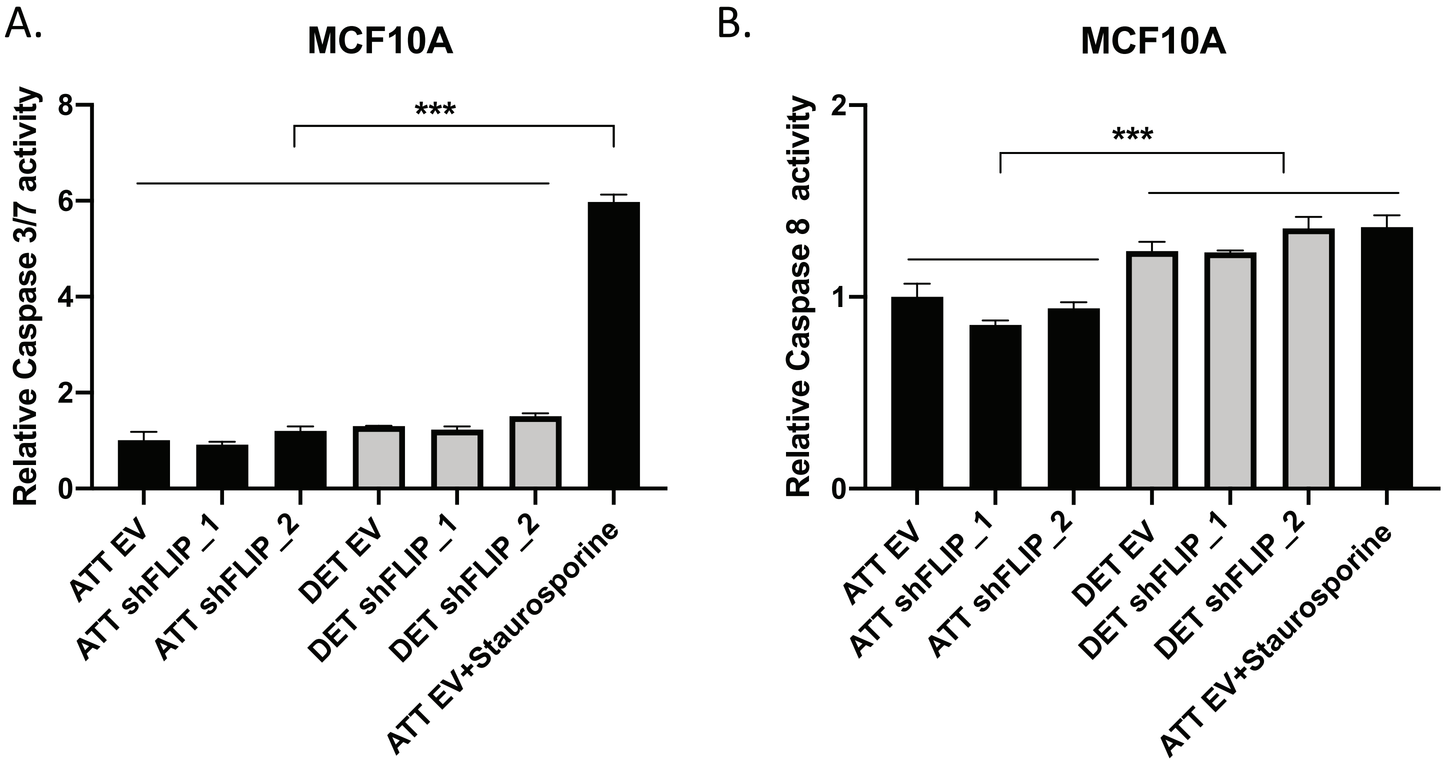

Supplemental Figure 4. shRNA-mediated reduction of C-FLIP$_L$ does not alter caspase activation in ECM-detached or ECM-attached cells.

Measurement of Caspase 3/7 activity (A) or Caspase 8 activity (B) in the indicated cells after being grown for 24h in ECM-detached conditions. Cells were treated with staurosporine as a positive control for caspase 3/7 activation. Statistical significance was determined using Student's two-tailed t-test. Error bars show standard deviation.

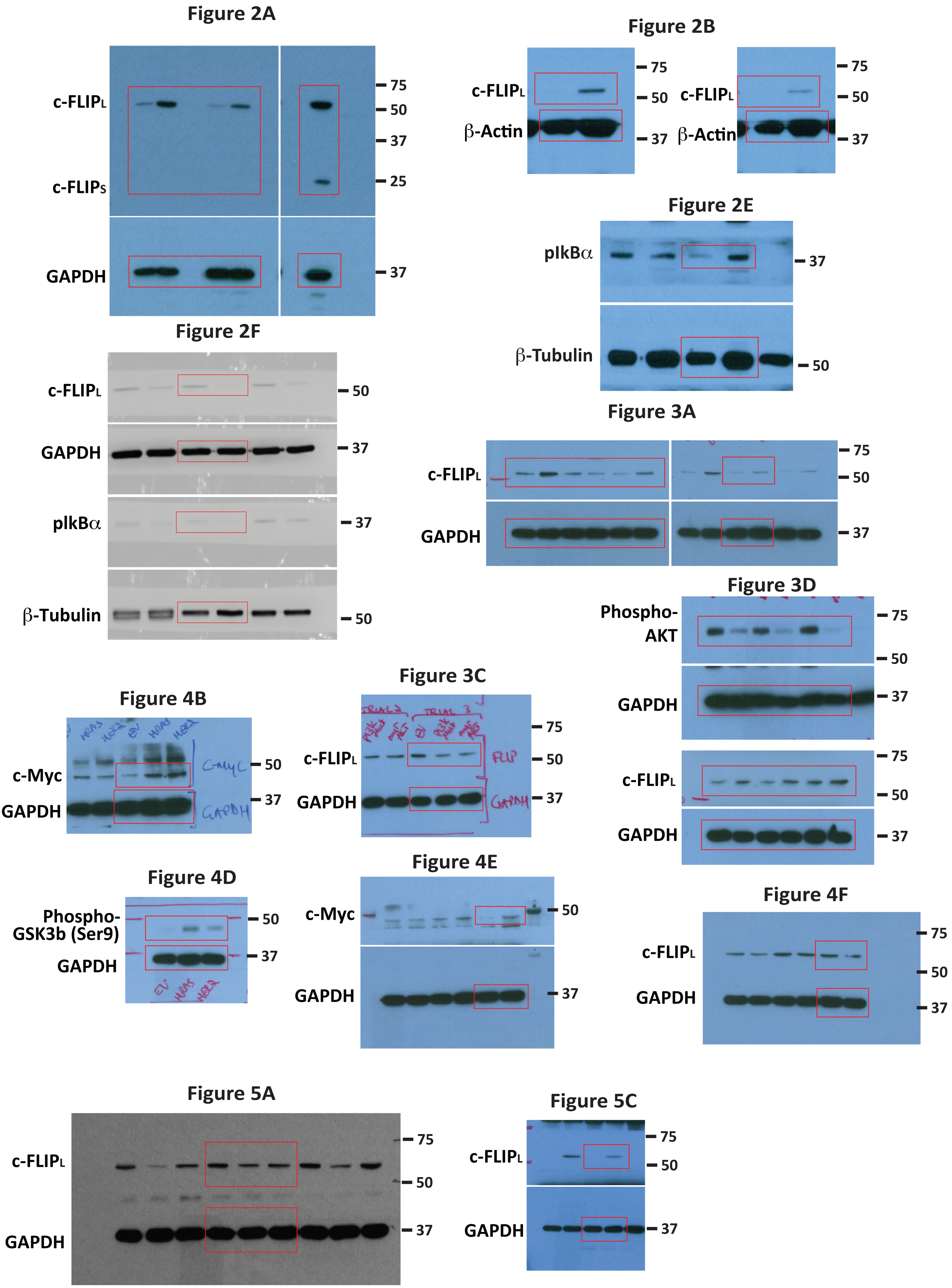

Supplemental Figure 5. Original, unprocessed data from Figures 1-5

Supplemental Figure 2A

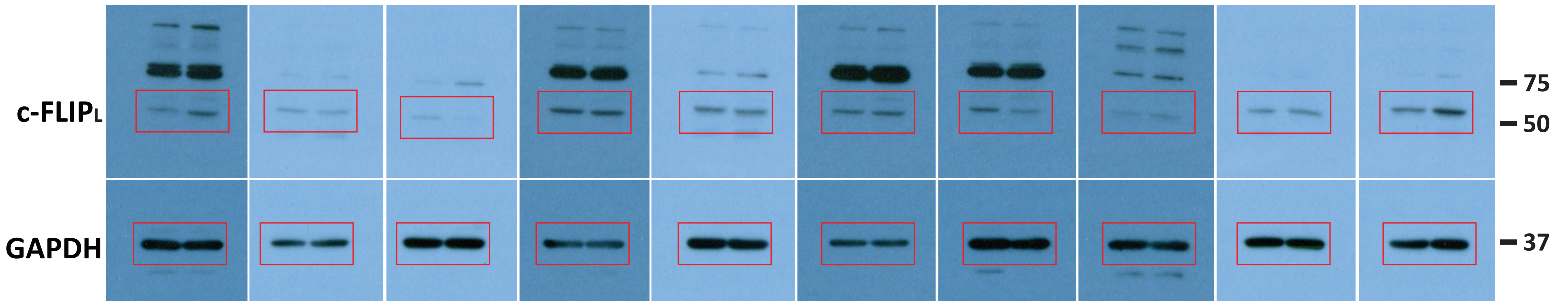

Supplemental Figure 2B

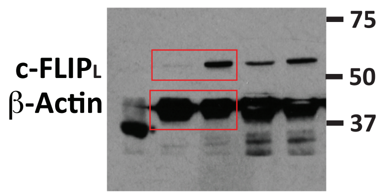

Supplemental Figure 3A

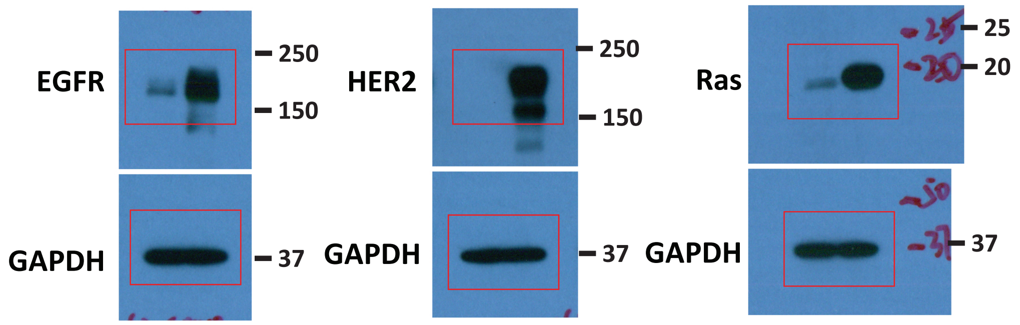

Supplemental Figure 3B

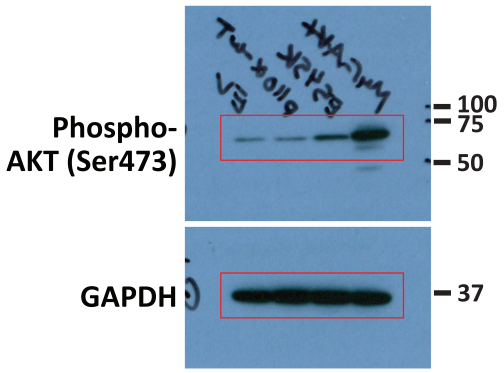

Supplemental Figure 6. Original, unprocessed data from Supplemental Figures 1-4
